# C-ter100 peptide derived from Vibrio vEP-45 protease acts as a pathogen-associated molecular pattern to induce inflammation and innate immunity

**DOI:** 10.1371/journal.ppat.1012474

**Published:** 2024-08-26

**Authors:** Jung Eun Park, Ji-Hye Yun, Weontae Lee, Jung Sup Lee

**Affiliations:** 1 Department of Biomedical Science, College of Natural Sciences and Public Health and Safety, Chosun University, Gwangju, Republic of Korea; 2 BK21-Four Educational Research Group for Age-associated Disorder Control Technology, Chosun University, Gwangju, Republic of Korea; 3 Department of Biochemistry, College of Life Science and Biotechnology, Yonsei University, Seoul, Republic of Korea; 4 Center for Genome Engineering, Institute for Basic Science (IBS), Daejeon, Republic of Korea; University of California Davis School of Medicine, UNITED STATES OF AMERICA

## Abstract

The bacterium *Vibrio vulnificus* causes fatal septicemia in humans. Previously, we reported that an extracellular metalloprotease, vEP-45, secreted by *V*. *vulnificus*, undergoes self-proteolysis to generate a 34 kDa protease (vEP-34) by losing its C-terminal domain to produce the C-ter100 peptide. Moreover, we revealed that vEP-45 and vEP-34 proteases induce blood coagulation and activate the kallikrein/kinin system. However, the role of the C-ter100 peptide fragment released from vEP-45 in inducing inflammation is still unclear. Here, we elucidate, for the first time, the effects of C-ter100 on inducing inflammation and activating host innate immunity. Our results showed that C-ter100 could activate NF-κB by binding to the receptor TLR4, thereby promoting the secretion of inflammatory cytokines and molecules, such as TNF-α and nitric oxide (NO). Furthermore, C-ter100 could prime and activate the NLRP3 inflammasome (NLRP3, ASC, and caspase 1), causing IL-1β secretion. In mice, C-ter100 induced the recruitment of immune cells, such as neutrophils and monocytes, along with histamine release into the plasma. Furthermore, the inflammatory response induced by C-ter100 could be effectively neutralized by an anti-C-ter100 monoclonal antibody (C-ter100Mab). These results demonstrate that C-ter100 can be a pathogen-associated molecular pattern (PAMP) that activates an innate immune response during *Vibrio* infection and could be a target for the development of antibiotics.

## Author summary

**Fig 1:**
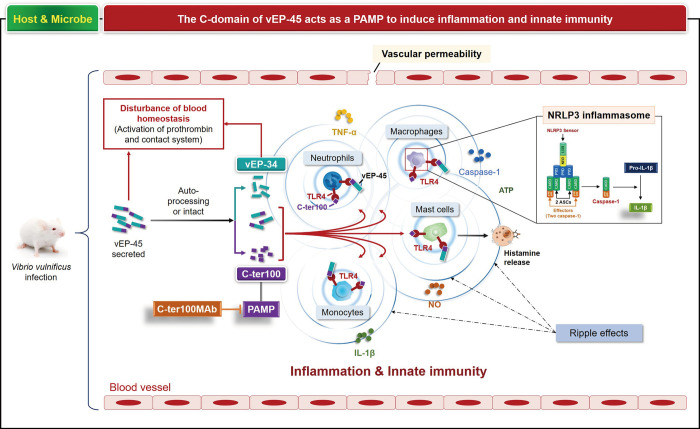
The C-domain of vEP-45 acts as a PAMP to induce inflammation and innate immunity. The diagram illustrates the mechanism by which the C-domain of vEP-45 induces inflammation and innate immunity in response to *Vibrio vulnificus* infection. Upon infection, vEP-45 is secreted and processed into vEP-34 and C-ter100 fragments, which function as pathogen-associated molecular patterns (PAMPs). These fragments bind to Toll-like receptor 4 (TLR4) on various immune cells. This binding leads to the activation of inflammatory pathways, contributing to the overall immune response.

This study focused on the specific molecules associated with the pathogenic *Vibrio vulnificus* that play a role in the initiation of the innate immune responses during infection. The C-domain of vEP-45 and the C-ter100 derived from vEP-45 act as the first signals (as PAMP) to prime the formation of NLRP3 inflammasome, during which they bind to TLR4, thereby activating it (as PRR), leading to the activation of NF-κB signaling pathway to produce a variety of inflammatory cytokines and regulators such as TNF-α and NO, accompanied with the expression of NLRP3 (Fig 1). The formation and activation of NLRP3 inflammasome are triggered by ATP, serving as the second signal and resulting in production of active caspase-1 (Casp 1), which converts pro-IL-1β into IL-1β. Anti-C-ter100 monoclonal antibody (C-ter100Mab) can relieve the inflammatory response by neutralizing both vEP-45 and C-ter100 (Fig 1). Conclusively, C-ter100 functioned as a novel pathogen-associated molecular pattern, potentiating the innate immune system, and thus constituting a new target for the development of antibiotics. We believe that our study makes a significant contribution to the literature because it confirmed the binding between TLR4 and C-ter100 that initiates the innate immune response against Vibrio infection and determined that blocking this binding with C-ter100Mab effectively inhibited vibriosis. These results are important because they further elucidated *Vibrio* pathogenesis and helped in the designing new treatments and therapies that could control vibriosis.

## Introduction

The innate immune system is the first line of defense against microbial pathogens. The system is operated via various pathogen recognition receptors (PRRs) that are activated by binding to distinct conserved structures from the pathogens, also known as pathogen-associated molecular patterns (PAMPs) [1–5]. For example, lipid A, an important component of lipopolysaccharide (LPS) in the outer membrane of Gram-negative bacteria, is a PAMPs detected by a PRR, Toll-like receptor 4 (TLR4) [2].

The marine bacterium *Vibrio vulnificus* (*V*. *vulnificus*), an opportunistic human pathogen, can cause systemic infection and severe sepsis, especially when people with immune disorders and liver problems consume raw seafood, such as oysters contaminated with bacteria [6–8]. Although *V*. *vulnificus*-mediated sepsis can be treated using a combination of drugs, such as cefepime, levofloxacin, and doxycycline, the mortality for patients reaches 40%–50% [9]. Even after treatment, sequelae and/or complications, such as redness, swelling, heat, pain, and loss of tissue function, often occur due to local immune, vascular, and inflammatory cell responses [10,11].

*V*. *vulnificus* secretes several extracellular virulence factors, such as RtxA (Vibrio toxin), VvhA (hemolysin), and VvpE (elastase) [12,13] that are known to induce apoptosis and necrosis of immune cells with various cytotoxic effects [12–17]. However, the exact role of these factors during *Vibrio* infection is poorly understood. Previously, we reported that *V*. *vulnificus* secretes an extracellular zinc-metalloprotease [18], which was secreted into the culture medium as a zymogen. Inactivated by its N-terminal pro-peptide domain [19], this proenzyme undergoes self-proteolysis to generate a catalytically-active 45 kDa enzyme (vEP-45) by losing the N-terminal propeptide domain [19]. vEP-45 is further autoprocessed into another active 34 kDa protease (vEP-34) by releasing an 11 kDa C-terminal peptide fragment (C-ter100) [18,19]. Previous studies have also revealed that vEP-45 and vEP-34 proteases disturb blood homeostasis by activating prothrombin [18] and the plasma contact system [20], which are related to their proteolytic activities. We also recently determined the three-dimensional structure of C-ter100 using NMR [21].

Although several studies have been published regarding the interaction between *Vibrio* strains and immune cells [22–24], the role(s) of the extracellular bacterial protease (and the fragments derived from the protease) in inducing innate immunity in terms of PRR and PAMPs is still unknown.

Herein, we report, for the first time that vEP-45’s C-domain and the C-ter100 peptide act as PAMP in activating NF-κB and the NLRP3 inflammasome by binding to TLR4, stimulating various inflammatory cytokines and recruiting immune cells to induce an innate immune response during bacterial infection.

## Results

### Molecular cloning and purification of vEPs, C-ter100, and N-ter139 peptides

**Fig 2:**
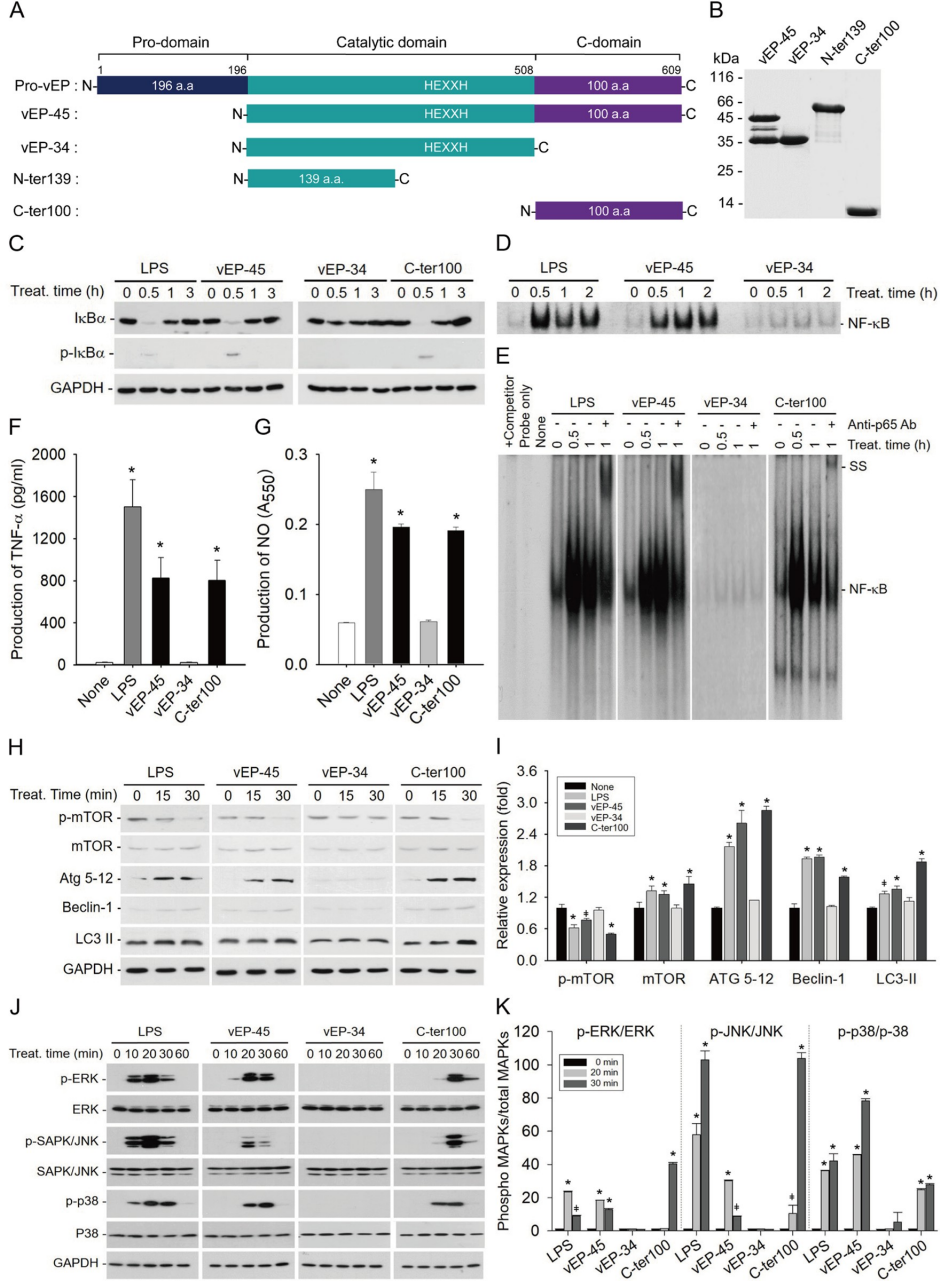
Activation of inflammatory response and autophagy by vEP-45 and C-ter100 in RAW264.7 cells. (A) Schematic organization of vEP proteases and its derived peptides. The inactive proenzyme vEP (pro-vEP) is composed of 609 amino acids and has three domains, including pro-domain, catalytic domain containing a Zn^2+^-binding motif (H^343^EXXH^347^), and C-domain. The pro-domain is released from pro-vEP to generate an active vEP-45 protease, which is further processed into vEP-34 and C-ter100. (B) Purification of vEP-45, vEP-34, MBP-tagged N-ter139, and C-ter100. The purified vEP-45, vEP-34, N-ter139, and C-ter100 were electrophoresed on a 12% SDS-polyacrylamide gel. (C) vEP-45 and C-ter100 induced degradation of IkBα. RAW264.7 cells were treated with 1 μg/ml each of LPS, vEP-45, vEP-34, and C-ter100 for various time periods as indicated and 50 μg each of cell lysates was subject to SDS-PAGE, and western blottings were conducted using anti-IκBα,–phosphorylated IκBα (p-IkBα), or -GAPDH antibodies. (D) EMSA for examining the activation of NF-κB by vEP-45 and C-ter100. Nuclear extracts were prepared from treated RAW264.7 cells as indicated. Ten μg each of nuclear extract was incubated with [γ-^32^P]-labeled consensus NF-κB binding element for 20 min. The resulting complex was separated on a 6% native polyacrylamide gel and autoradiographed on an X-ray film. (E) Supershift assay for examining the translocation of NF-κB into the nucleus by vEP-45 and C-ter100. Ten μg of nuclear extracts were combined with [γ-32P]-labeled consensus NF-κB binding element and anti-NF-κB p65 antibodies, followed by co-incubation for 1 hour at room temperature. The resulting complexes were then separated on a 6% native polyacrylamide gel and visualized by autoradiography on X-ray film. Super-shifted bands (SS) were observed, indicating the presence of NF-κB bound to the labeled element. Additionally, a control sample was incubated with cold NF-κB binding element as a competitor. (F) Production of TNF-α by vEP-45 and C-ter100. Raw 264.7 cells were exposed to LPS, vEP-45, vEP-34, and C-ter100 at a concentration of 1 μg/ml for 3 hours. Subsequently, the concentration of TNF-α in the culture supernatant was quantified using ELISA. The reported values represent the mean ± standard deviation (SD) derived from three independent experiments. (G) Production of NO by vEP-45 and C-ter100. Raw 264.7 cells were treated with 1 μg/ml each of protein 18 h and NO level in the culture medium was measured using Griess reagents as described in Materials and Methods. Results represent the mean ± SD of three independent experiments. (H to K) Activation of autophagy (H and I) and MAPK (J and K) signaling pathways by vEP-45 and C-ter100. RAW264.7 cells were treated with 1 μg/ml each of LPS, vEP-45, vEP-34, and C-ter100 for the indicated time periods and the cell lysates were separated by SDS-PAGE, followed by Western blottings with corresponding antibodies. The relative fold expression levels of p-mTOR, mTOR, Atg5, Beclin-1, LC3 II at 30 min are shown from two independent experiments, which are normalized to those of GAPDH (H and I). An average of incubation time of 0 was used as none. The p-ERK/ERK, p-JNK/JNK, p-p38/p38 ratios are shown from two independent experiments, which are normalized to incubation time 0 (J and K). The data in F, G, I, and K are representative of significant differences (*p < 0.005; ^ǂ^*p <* 0.05, significant differences compared to incubation time 0 using one-way ANOVA, followed by Dunnett’s multiple comparison test).

vEP-45- and vEP-34-encoding genes from the genomic DNA of ***V*. *vulnificus*** ATCC29307 were cloned and expressed in *E*. *coli*. The vEP-45 and vEP-34 proteases were purified as described previously [18, 19], and their overall schematic structures are shown in Fig 2A. The proenzyme vEP (pro-vEP), composed of 609 amino acids, is organized into three domains, including a pro-domain, a catalytic domain containing a Zn^2+^-binding motif (HEXXH), and a C-domain [18, 19]. The pro-domain is removed from the pro-vEP during its extracellular secretion, and a catalytically active 45 kDa-sized protease (named vEP-45) was generated [19]. The vEP-45 protease can be further processed to produce 34 kDa protease (named vEP-34) with reduced activity by releasing its C-domain (termed C-ter100). Here, the C-ter100- and N-terminal 139 amino acids-encoding (N-ter139) regions of the vEP-45 gene were PCR-amplified, expressed in *E*. *coli*, and purified as described in Materials and methods. The purification steps of C-ter100 and N-ter139 are summarized in S1 Table. The apparent molecular weights of purified vEP-45, vEP-34, C-ter100, and N-ter139 (15.3 kDa) fused to maltose-binding protein (MBP; 42.5 kDa) were approximaterly 45, 34, 12, and 58 kDa, respectively, based on the SDS-PAGE results (Fig 2B). The purified N-ter139 peptide was used as an antigen to produce polyclonal anti-vEP-45 antibody as described in Materials and methods.

### The C-domain of vEP-45 is involved in the induction of an inflammatory response

The inflammatory response caused by vEP-45, NF-κB activation through IκBα degradation was analyzed in RAW264.7 cells. Western blotting results showed the phosphorylation and subsequent degradation of IκB in cells treated with LPS (used as positive control), vEP-45, and C-ter100, but not vEP-34 (Fig 2C). These results suggested that the C-domain of vEP-45 can stimulate IκB degradation. After IκB degradation, NF-κB is released from the cytoplasmic IκB-NF-κB complex and then translocated into the nucleus [25]. After translocation, NF-κB binds to the upstream regions of target genes, increasing their transcription levels [26,27]. Electrophoretic mobility shift assay (EMSA) was performed to confirm this with γ-^32^P- labeled synthetic consensus sequence containing NF-κB binding site as a probe (Fig 2D). The EMSA data showed a mobility shift of the probe in the nuclear extracts prepared from the cells treated with LPS and vEP-45 but not in vEP-34-treated cells (Fig 2D). These results suggested that the C-domain of vEP-45 could be involved in the nuclear translocation of NF-κB, which was further evaluated by supershift assay with an anti-p65 antibody that can recognize an NF-κB component, p65. As shown in Fig 2E, supershifted (SS) bands appeared in the nuclear extracts prepared from LPS, vEP-45, and C-ter100-treated cells, demonstrating that the C-domain of vEP-45 activated NF-κB via IκB degradation. Based on these results, we used RT-PCR to examine the effects of vEP-45 and C-ter100 on the transcription levels of pro-inflammatory cytokines, including *Tnf-α*, *Il-1β*, and *Il-6*, in RAW264.7 cells. As shown in S1A–S1D Fig, *Tnf-α*, *Il-1β*, and *Il-6* mRNA levels were significantly elevated by LPS, vEP-45, and C-ter100 treatments but not by vEP-34. ELISA data also clearly showed that only the cells treated with vEP-45 and C-ter100 produced TNF-α, but not the ones treated with vEP-34 (Fig 2F).

Nitric oxide (NO) is produced by inducible NO synthase (iNOS), and prostaglandin Es (PGEs) are generated by cyclooxygenase-2 (Cox-2), both of which are involved in inflammatory processes. Specifically, prostaglandin E2 is synthesized when arachidonic acid is metabolized by Cox-2 into PGH2, and subsequently processed by one of the three types of prostaglandin E synthases, notably microsomal prostaglandin E synthase-1 (mPGEs-1) [28, 29]. Additionally, a chemokine macrophage inflammatory protein-2 (MIP-2) amplifies the inflammatory response by recruiting neutrophils [26]. Significant amounts of NO were produced in RAW264.7 cells treated with LPS, vEP-45, and C-ter100 but not vEP-34 (Fig 2G). Moreover, the RT-PCR results showed that the transcription levels of *Cox-2*, *Pges*, *iNos*, and *Mip-2* were also significantly upregulated in cells treated with LPS, vEP-45, and C-ter100, but not by, vEP-34 (S1E–S1I Fig). These results strongly suggested that the C-domain of vEP-45 was important for initiating and amplifying inflammatory responses.

Conversely, the induction and modulation of inflammation is associated with the activation of autophagy and MAPK signaling [30]. Therefore, we evaluated the effect of vEP-45, vEP-34, and C-ter100 on autophagy and MAPK signaling. Western blotting results showed that p-mTOR expression was down-regulated in the RAW264.7 cells treated with LPS, vEP-45, and C-ter100, while the expression of mTOR, ATG5-12, Beclin-1, and LC3-II was increased (Fig 2H and 2I). However, these effects were not observed with the vEP-34 treatment. Moreover, vEP-45 and C-ter100, but not vEP-34, could activate the MAPK signaling pathway through the phosphorylation of ERK, JNK, and p38 (Fig 2J and 2K). These results suggested that the C-domain of vEP-45 and C-ter100 peptide derived from the protease were involved in inducing and regulating inflammatory responses by triggering the activation of autophagy through the MAPK signaling pathway.

### The C-domain of vEP-45 binds to TLR4 to induce an inflammatory response

**Fig 3:**
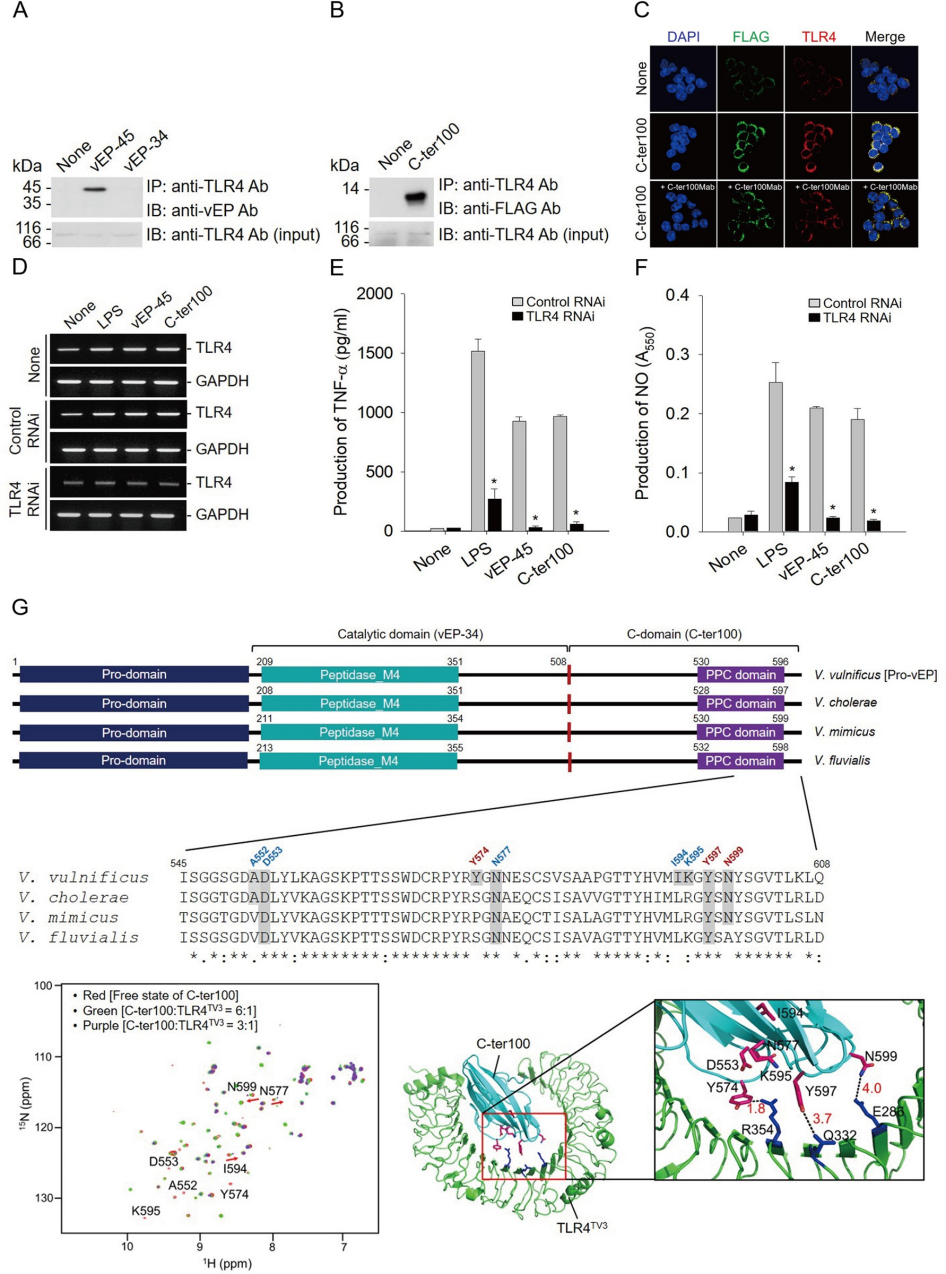
Binding of vEP-45 to TLR4 through its C-domain. (A) Immunoprecipitation (IP) and immunoblotting (IB) to show the binding of vEP-45 to TLR4. Anti-TLR-4 antibody-bound agarose beads were incubated overnight at 4°C with 50 μg of the membrane fraction of Raw 264.7 cells and 1 μg of vEP-45 or vEP-34. After separating the precipitated proteins using SDS-PAGE and Western blotting was performed with anti-vEP and -TLR4 antibodies, as indicated. ‘None’ refers to the data from untreated cells. (B) IP and IB to show the binding of C-ter100 to TLR4. Anti-TLR4 antibodies coupled to agarose beads were incubated overnight at 4°C with 50 μg of the membrane fraction of Raw 264.7 cells and 1 μg of C-ter100. The immunioprecipitated proteins were separated by SDS-PAGE and Western blottings were performed with anti-TLR4 and–FLAG antibodies as indicated. None means data from non-treated cells. (C) Immunofluorescence imaging of the binding of TLR4 and C-ter100 by confocal microscopy. RAW264.7 cells treated with C-ter100 (1 μg) in the presence or absence of C-ter100Mab (0.25 mg/ml) for 30 minutes were incubated overnight with anti-TLR4 or -FLAG antibodies, stained with rhodamine- or fluorescein-conjugated IgG antibodies for 1 h, respectively, and then observed with confocal microscope, in which the cells were also stained with DAPI to show nuclei. None means data from non-treated cells. (D) Expression of TLR4 receptor by vEP-45 and C-ter100. RAW264.7 cells transfected with control RNAi or TLR4-RNAi were treated with 1 μg each of LPS, vEP-45 or C-ter100 for 24 h and then RT-PCRs were performed with a pair of TLR4 primers. None means data from non-transfected cells. (E and F) Effect of RNAi on the production of TNF-α and NO. RAW264.7 cells transfected with control RNAi or TLR4-RNAi were treated with 1 μg each of none, LPS, vEP-45 or C-ter100 for 00 h, from which the amounts of TNF-α (E) and NO (F) were measured as described in Fig 1F and 1G. ***p*-**value was obtained using a paired *t*-test (*p < 0.001 compared with each treated group). (G) Mapping of the binding of C-ter100 to TLR4 receptor by NMR. (Top panel) Comparison of domain structures of proteases secreted by four Vibrio strains and alignment of amino acid sequences between their C-terminal regions. Eight amino acid residues located in the C-terminal region of vEP-45 that can interact with TLR4, identified by NMR studies, are shown in the amino acid sequence. (Bottom left panel). Molecular interaction between C-ter100 and TLR4^TV3^, for which NMR titration was carried out by combining unlabeled TLR4^TV3^ with ^15^N-labeled C-ter100 at two molar ratios of 3:1 and 6:1 to identify the binding sites. (Bottom right panel) Molecular docking between C-ter100 (Cyan) and TLR4^TV3^ (green), for which the complex model was established by the AutoDock program with human TLR4 (PDB ID: 4G8A) as a template. The residues that participate in the binding are shown in the stick model (C-ter100, magenta; TLR4^TV3^, blue). More detailed binding sites between C-ter100 and TLR4 are shown in the enlarged box.

Toll-like receptors (TLRs) are crucial for inducting innate immunity by sensing invading microorganisms [31]. Therefore, the binding ability of vEP-45 to TLR4 was examined using co-immunoprecipitation (Co-IP) and immunofluorescence assay (Fig 3A–3C). The Co-IP and Western blotting results showed that vEP-45 and C-ter100 precipitated with TLR4 but not vEP-34 (Fig 3A–3B). These results were further confirmed by examining the colocalization of C-ter100 and TLR4 on the cell surface using immunostaining with confocal microscopy, using fluorescein- and rhodamine-conjugated secondary antibodies to visualize C-ter100 and TLR4, respectively. C-ter100 and TLR4 were co-localized on the cell surface (Fig 3C), demonstrating that vEP-45 binds to TLR4 on the cell surface through its C-domain. Additionally, to assess the inhibitory ability of C-ter100Mab on the binding of C-ter100 to macrophages, we treated the cells with C-ter100Mab. The results showed that treatment with C-ter100Mab significantly inhibited the binding of C-ter100 to macrophages (Fig 3C), suggesting that this interaction is specific to the C-ter100 peptide.

The effect of vEP-45 or C-ter100 on TLR4 expression was investigated using RT-PCR in RAW264.7 cells. Cells treated with 1 μg/ml of vEP-45 or C-ter100 for 30 min exhibited significantly upregulated TLR4 gene expression (1.60- and 1.64-fold increase, respectively) compared to the nontreated control (two top panels, Fig 3D). However, when RAW264.7 cells transfected with TLR4 RNA interference (RNAi) were treated with the same concentrations of vEP-45 and C-ter100 for 30 min, the TLR4 level was not increased, compared to those of mocked cells (two down panels, Fig 3D). This data revealed that TLR4 gene expression could be upregulated by the C-domain of vEP-45. Moreover, the cells transfected with RNAi for TLR4 produced significantly low levels of TNF-α (Fig 3E) and NO (Fig 3F) when treated with vEP-45 and C-ter100. When the TLR4 RNAi-transfected cells were incubated with 1 μg/ml each of vEP-45 and C-ter100 for 3 h, TNF-α production decreased by 96.3% and 94.05% (*p* < 0.005), respectively, compared with those of the non-transfected cells (Fig 3E). Under the same experimental conditions, the NO production was also reduced by vEP-45 and C-ter100 to 88.6% and 90.3% (*p* < 0.005), respectively (Fig 3F). Thus, TLR4 is crucial for inducing vEP-45-mediated inflammatory response by binding to the C-domain of the protease.

### Three amino acid residues of C-ter100 participate in binding to the cell surface receptor TLR4

Previously, we reported that using NMR, C-ter100 forms a β-barrel structure containing eight β-strands [21]. C-ter100 also has a PPC domain that is highly conserved in various extracellular proteases from *Vibrio* strains, including *V*. *cholerae*, *V*. *mimicus*, and *V*. *fluvialis* (Top panel, Fig 3G). In this study, the binding sites of C-ter100 to TLR4 were determined by NMR titration and molecular docking (Fig 3G). NMR titration results showed that binding with TLR4 caused a significant chemical shift perturbation in three amino acid residues (Asn^577^, Ile^594^, and Asn^599^) located in the loop region of the PPC domain of C-ter100. Notably, the spectra of four residues (Ala^552^, Asp^553^, Tyr^574^, and Lys^595^) exhibited a spectral line broadening upon TLR4 binding, suggesting that they were also involved in the interaction with TLR4 (bottom left panel, Fig 3G). Based on these results, the complex model between C-ter100 and TLR4 was built using the AutoDock program. The molecular docking results showed that Arg^354^ and Gln^332^ in TLR4^TV3^ participated in the cation-π interactions with Tyr^574^ and Tyr^597^ residues of C-ter100. The modeled structure also revealed a carbonyl-carbonyl interaction between Glu^286^ of TLR4 and Asn^599^ of C-ter100 (bottom right panel, Fig 3G). These findings indicated that vEP-45 directly interacted with TLR4 through three residues (Tyr^574^, Tyr^597^, and Asn^599^) located in the C-domain.

### The C-domain of vEP-45 and the C-ter100 can induce the formation and activation of NLRP3 inflammasome

**Fig 4:**
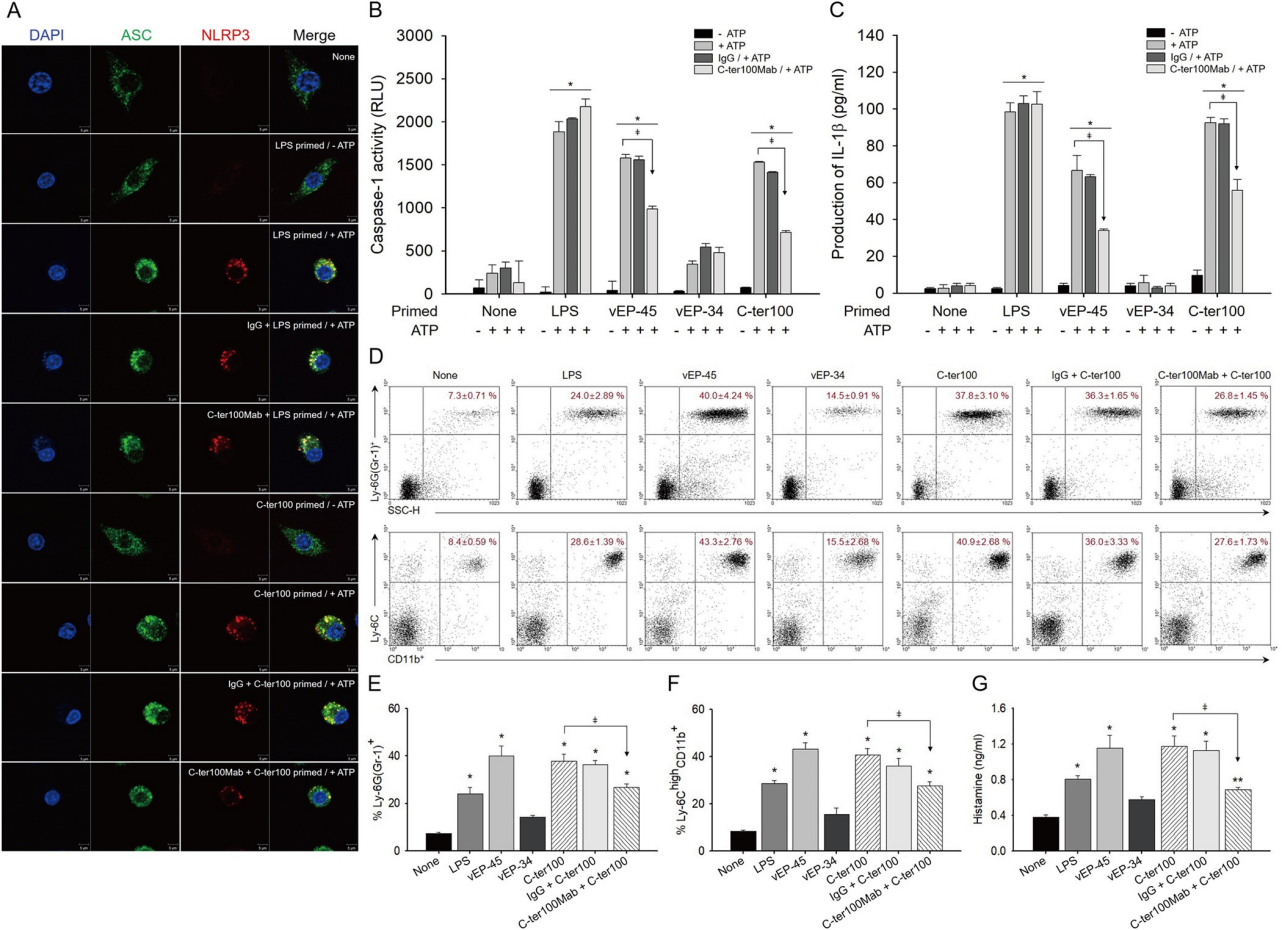
Formation of NLRP3 inflammasome, recruitment of immune cells, and release of histamine release by vEP-45 and C-ter100. (A) Immunofluorescence detection of NLRP3 and ASC in BMDMs treated with LPS and C-ter100 by confocal microscopy. BMDMs were none-treated or primed with LPS and C-ter100 (0.1 μg) for 3 h in the absence or presence of mouse IgG and anti-C-ter100 monoclonal antibody (C-terMab) and then none or stimulated with 2 mM of ATP for 2 h at 37°C. The cells were fixed, immuno-stained overnight with anti-ASC antibody coupled to fluorescein or anti-NLRP3 conjugated with rhodamine as indicated, and observed with confocal microscope. The cells were also stained with DAPI to show the nuclei. (B and C) Production of caspase-1 and IL-1β by vEP-45 and C-ter100. BMDMs were none-treated or primed with LPS, vEP-45, vEP-34, and C-ter100 (1 μg/ml) for 3 h in the absence or presence of mouse IgG and anti-C-ter100 monoclonal antibody (C-terMab) and then none or stimulated with 2 mM of ATP for 2 h, as indicated, from which the caspase 1 activity was measured using a Caspase-Glo 1 inflammasome assay kit (B) and the IL-1β produced was determined using an ELISA kit (C). "+" and "-" mean treated and untreated group, respectively. Histograms show the mean values (±SD) from three-independent experiments. (D-F) FACS analysis of immune cells in mice treated with vEP-45 and C-ter100. BALB/c mice (n = 6/group) were injected with 2.5 mg/kg of LPS, vEP-45, vEP-34, and C-ter100 via the tail vein. After collecting whole blood 3 h later RBCs were lysed, and the remaining cells were used for FACS analysis with antibodies raised against Ly-6G^+^, Ly-6C^+^, and CD11b^+^. The Ly-6G(Gr-1)^+^ and Ly-6C^high^CD11b^+^ cells are shown in the upper right quadrants (D). Sing cells were gated from total cells based on their forward and side scatter. The portions of Ly-6G(Gr-1)^+^ (E; neutrophils) and Ly-6C^high^CD11b^+^ (F; monocyte) cells were calculated and expressed as percentages in the gated regions. (G) Production of histamine by vEP-45 and C-ter100 in mice. BALB/c mice (n = 6/group) were injected with 2.5 mg/kg each of LPS, vEP-45, vEP-34, and C-ter100 via tail veins. After collecting whole bloods 3 h later, the plasma histamine concentrations were measured using an ELISA. The data in B, C, E, F, and G are representative of three-independent experiments (*, *p* < 0.001; **, *p* < 0.01; ^ǂ^, *p* < 0.05; significant differences compared to that of the untreated sample, which was calculated with one-way ANOVA, followed by Dunnett’s multiple comparison test; ***p*-**values were obtained using a paired *t*-test, compared with the group indicated).

NLRP3 (NOD-, LRR-, and pyrin domain-containing protein 3) is an intracellular sensor that activates the NLRP3 inflammasome by detecting various microbial-derived substances and cellular danger signals [32]. When triggered, the NLRP3 inflammasome induces caspase 1-dependent release of the pro-inflammatory cytokine IL-1β [33]. The role of vEP-45 and C-ter100 in the formation and activation of the NLRP3 inflammasome was investigated using BMDMs primed with LPS (as a positive control) or C-ter100, followed by ATP stimulation (Fig 4A). For confocal microscopy, the cells were stained with an anti-ASC antibody coupled with fluorescein, an anti-NLRP3 antibody conjugated to rhodamine, and DAPI to show nuclei. The NLRP3 inflammasomes were only found in the cells primed with LPS and C-ter100, followed by ATP stimulation (Fig 4A). These results suggested that C-ter100 might be involved in forming the NLRP3 inflammasome, which can be activated by ATP stimulation. Furthermore, caspase-1 activity assay and IL-1β ELISA showed that LPS, vEP-45, and C-ter100 increased caspase-1 activity by 2.01-, 1.83-, and 1.80-fold (Fig 4B) and IL-1β production by 39.32-, 26.61-, and 37.01-fold (Fig 4C), respectively, only in ATP-stimulated BMDMs, compared to that of non-treated control. However, vEP-34 could not increase caspase-1 activity and IL-1β production (Fig 4B–4C). All these results suggested that vEP-45, through its C-domain, could prime the formation of NLRP3 inflammasomes to be activated by ATP, thereby significantly increasing caspase-1 activity and IL-1β production.

### vEP-45 and C-ter100 peptides can induce immune cell recruitment and histamine release

The ability of vEP-45 and C-ter100 to recruit immune cells, mainly neutrophils and monocytes, was investigated using *in vivo* flow cytometry (FACS) (Fig 4D–4F). For FACS analysis, PBS, LPS, vEP-45, vEP-34, or C-ter100 were injected into the tail veins of mice, and the blood was collected 3 h later. This was further used to prepare RBC-lysed blood cells. When the cells collected were analyzed, Ly-6G(Gr-1)^+^ cells, such as neutrophils, accounted for 7.3% in PBS-injected control. However, the cell counts for LPS, vEP-45, vEP-34, and C-ter100 treated samples were 24, 40, 14.5, and 37.8%, respectively (upper panels in Fig 4D–4E). These results suggested that vEP-45 and C-ter100 peptides could significantly increase the number of neutrophils. Similar results were also obtained for monocytes: LPS, vEP-45, vEP-34, and C-ter100 increased Ly-6C^high^CD11b^+^ monocytes by 28.6, 43.3, 15.5, and 40.9%, respectively (lower panels in Fig 4D and 4F). These findings demonstrate that vEP-45’s C-domain and vEP-45-derived C-ter100 peptide were involved in recruiting the immune cells, including neutrophils and monocytes, to potentiate innate immunity.

Histamine produced by immune cells, such as mast cells, basophils, and neutrophils, dilates blood vessels and recruits the immune cells to respond to foreign pathogens [34–36]. Histamine, an inflammatory mediator, also triggers the production of proinflammatory cytokines and regulators, such as IL-6 and TNF-α, NO, to stimulate inflammatory response [35, 37]. Therefore, the effects of vEP-45 and C-ter100 on histamine production were examined. LPS, vEP-45, and C-ter100 increased histamine production (by 2.12-, 3.04-, and 3.09-fold, respectively) compared to non-injected control (Fig 4G). However, the histamine levels did not change significantly after the vEP-34 treatment. These results suggested that the C-domain of vEP-45 and C-ter100 could induce histamine release, further enhancing immune cell recruitment.

### Anti-C-ter100 monoclonal antibody (C-ter100Mab) can neutralize C-ter100-induced inflammatory response and innate immunity

To study the effects of a monoclonal antibody raised against the C-ter100 peptide (C-ter100Mab) on C-ter100 peptide-induced TNF-α production in RAW264.7 cells (S2 Fig), NLRP3 inflammasome formation in BMDMs (Fig 4A–4C), immune cell recruitment (Fig 4D–4F), and histamine release in mice (Fig 4G). C-ter100Mab significantly reduced the C-ter100-induced TNF-α production by 39.58%, while control mouse IgG (IgG) treatment decreased it by only 2% in RAW264.7 cells (S2 Fig). C-ter100Mab also decreased the C-ter100-induced formation of NLRP3 inflammasome in BMDM, as observed with confocal microscopy (Fig 4A). Further, C-ter100Mab reduced C-ter100-induced caspase-1 activity (Fig 4B) and IL-1β production (Fig 4C) by 53.37 and 39.9%, respectively, compared to those by control untreated C-ter100Mab. FACS data also showed that C-ter100Mab could decrease C-ter100-induced increases of neutrophil and monocyte counts by an average of 31.22%, compared to C-ter100 alone (Fig 4D–4F). C-ter100Mab also exhibited a strong neutralizing activity by decreasing the C-ter100-induced histamine release by 60.93%, compared to C-ter100 alone in mice (Fig 4G). These results suggest that the C-ter100Mab can effectively neutralize C-ter100-induced inflammation and innate immunity.

## Discussion

The pathogenesis of Vibrio spp. infection, leading to inflammation and septic shock [38], in relation to the extracellular bacterial protease vEP-45 is not fully understood. The enzyme’s broad specificity [18] and diverse biological activity [19,39] suggest its potential as a virulence factor secreted by *V*. *vulnificus*. In this study, we aimed to investigate the role of C-ter100, a fragment derived from vEP-45 as a PAMP that induces inflammation and innate immunity in *Vibrio* pathogenesis.

To confirm the induction of an immune response by PAMPs derived from *V*. *vulnificus*, such as vEP-45 and its processed fragment C-ter100, our study was conducted. The result revealed the role of vEP-45 and C-ter100 in enhancing the production of pro-inflammatory cytokines, TNF-α, and NO (Fig 2F–2G) through NF-*κ*B activation (Fig 2C–2E). These findings indicate that vEP-45 and C-ter100 act as PAMPs, triggering inflammatory responses during Vibrio infections.

TLRs are primarily expressed in immune cells and play a crucial role in recognizing PAMPs for the innate immune response [40]. Therefore, we revealed the direct binding between C-ter100 (as PAMPs) and TLR4 (as PRRs) through CO-IP analysis (Fig 3A–3B), and the binding sites were identified through NMR titration (Fig 3G). The structure of C-ter100 is known [21], and our NMR titration findings demonstrate three specific binding sites between C-ter100 and TLR4 (Fig 3G). Notably, these binding residues are conserved in human infectious Vibrio strains, including *V*. *vulnificus*, *V*. *cholerae*, and *V*. *mimicus* (Fig 3G). Taken together, these finding suggest that C-ter100 may act as a PAMP by recognizing TLR4 during Vibrio infection.

Inflammation is initiated by various immune cells such as macrophages, neutrophil, dendritic cells, histiocytes, kupffer cells, and mast cells [41]. At the onset of infection, these cells are activated as their PRRs recognize PAMPs, leading to the release of inflammatory mediators [11,42,43]. The reason for focusing on inflammasomes and neutrophil/monocyte numbers is their critical role as the first responders in innate immune responses to infections. They are recruited to the site of infection to perform phagocytosis, which removes pathogens, and to release cytokines, which orchestrate further immune responses. Therefore, the observed increase in neutrophil and monocyte numbers following C-ter100 administration directly suggests an enhanced immune response, crucial for understanding the body’s combats mechanisms against *V*. *vulnificus*.

**Fig 5:**
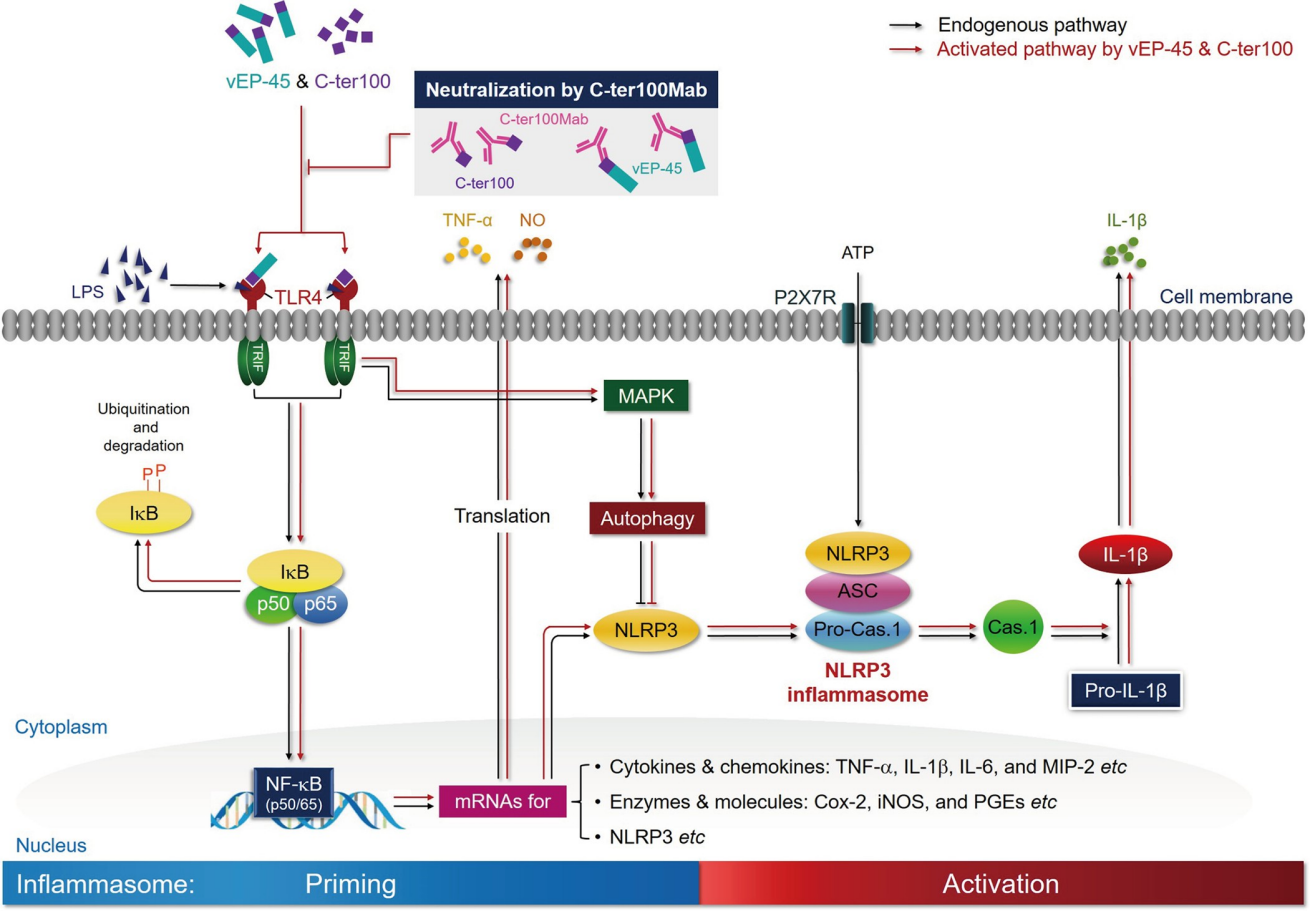
vEP-45- and C-ter100-induced signaling pathways involved in activating NLRP3 inflammasome. The C-domain of vEP-45 and the C-ter100 derived from vEP-45 act as the first signals (as PAMP) to prime the formation of NLRP3 inflammasome, during which they bind to TLR4 to be activated (as PRR), leading to the activation of NF-κB signaling pathway to produce a variety of inflammatory cytokines and regulators such as TNF-α and NO, accompanied with the expression of NLRP3. The formation and activation of NLRP3 inflammasome is triggered by ATP as the second signal, producing an active caspase-1 (Cas.1) to make IL-1β from pro-IL-1β. Anti-C-ter100 monoclonal antibody (C-ter100Mab) can relieve the inflammatory response by neutralizing both vEP-45 and C-ter100. In response to vEP-45 and C-ter100, the immune cells also activate autophagy to reduce inflammation by inhibiting NLRP3.

PAMPs recognition leads to transcriptional and post-transcriptional downstream responses [33,44]. The NLRP3 inflammasome, a key player in the post-translational inflammasome response, is activated following TLR signaling [32,33]. After TLR signaling initiates pro-IL-1β and pro-IL-18 transcription, the post-translational inflammasome response subsequently causes the cleavage and secretion of the active cytokines [24, 44, 45]. In the case of macrophage infection by *V*. *vulnificus* and *V*. *cholerae*, the NLRP3 inflammasome senses the infection, leading to caspase-1 activation and IL-1β secretion [24]. Interestingly, C-ter100-priming also induces NLRP3 inflammasome formation (Fig 4A), followed by IL-1β secretion (Fig 4C) and caspase 1 activation (Fig 4B), demonstrating a post-translational inflammasome response. vEP-45 and C-ter100 induced a significantly greater increase in the number of neutrophils/monocytes than LPS (Fig 4D–4F). Furthermore, C-ter100Mab significantly neutralized these effects *in vitro* and *in vivo* (Figs 4 and 5). It showed a reduction in TNF-α *in vitro* and reduced immune cell recruitment *in vivo*, although this reduction was rather modest. Therefore, these results suggested that C-ter100 is a novel PAMP and that C-ter100Mab effectively inhibited vibriosis.

## Conclusion

In conclusion, this study provides evidence for the role of C-ter100, derived from vEP-45 in the Vibrio infection process, in recognizing TLR4 and influencing innate immunity. C-ter100 act as primary signals (as PAMPs) initiating NLRP3 inflammasome formation through TLR4 (as PRRs) activation and NF-κB pathway induction, leading to the release of inflammatory mediators. The neutralizing effect of C-ter100Mab on vEP-45 and C-ter100 further alleviates inflammation and innate immune responses (Fig 5). The identifying the homeodomains such as C-ter100 among *Vibrio*-derived substances that induce an inflammatory response might help elucidate mechanisms underlying *Vibrio* infection, facilitating the design and development of the first discovery treatment options. However, utilizing the C-ter100 mutagenesis strategy to identify key TLR4-binding residues and investigate their function in diverse Vibrios along with the generation of single and multiple deletion mutants is important for a comprehensive understanding of its role in the complex pathogenicity of *V*. *vulnificus*. Furthermore, studies on macrophages infected with wild-type *V*. *vulnificus* and C-ter100 or vEP-45 deletion mutants and mouse infection models will help to elucidate the important involvement of C-ter100 peptide in inducing inflammation and influencing sepsis progression. It remains a crucial focus for future research to improve therapeutic strategies for Vibrio infections.

## Materials and methods

### Ethics statement

All animal experiments were performed under the approval of Chosun University Institution Animal Care and Use Committee (IRB #: CIACUC2019-A0016-1).

### Culture of bacterial and mammalian cells

E. coli DH5α and *V*. *vulnificus* ATCC29307 were cultured in LB medium with 0.5% and 1% NaCl at 37°C. RAW264.7 cells from ATCC (Manassas, VA, USA) were cultured in DMEM supplemented with 10% FBS and 1% penicillin/streptomycin at 37°C under 5% CO_2_.

### Isolation of Bone marrow-derived macrophage (BMDMs)

Male 6-week-old BALB/C mice were obtained from Orient Bio (Seoul, Korea), from which BMDMs were isolated from their femurs and tibia as described elsewhere [46–48]. The cell suspension prepared from the mice was treated with RBC lysis buffer (eBioscience, San Diego, USA) and centrifuged at 1,500 x*g* for 4 min. The cell pellet was suspended in DMEM and filtered through a cell strainer (40 μm in pore size).

### Cloning and purification of vEPs and C-ter100

vEP-45- and vEP-34-encoding genes were cloned and expressed in *E*. *coli*, from which vEP-45 and vEP-34 proteases were purified as described previously [19]. Subsequently, the C-ter100 gene was PCR-amplified from the genomic DNA of *V*. *vulnificus* ATCC29307 using the forward primer 5′-GTCAAGCTTAATGTGTTGAAAAACAACACGCCA-3′ and the reverse primer 5′-CGGGGTACC TCAATATTGCAGCTTTAA-3′, with the underlined bases indicating the introduced restriction sites, HindIII and KpnI, respectively. Amplification was carried out using the Applied Biosystem 9700 thermal cycler with the following conditions: 30 cycles of 94°C for 30 s, 50°C for 30 s, and 72°C for 30 s. The PCR product was digested with HindIII and KpnI, and then ligated with HindIII/KpnI-cleaved pFLAG-ATS vector to generate the recombinant plasmid pC-ter100 containing the C-ter100-encoding gene.

To express the C-ter100, *E*. *coli* DH5α cells containing pC-ter100 were cultured overnight in LB broth with ampicillin (100 μg/ml). Ten ml of the overnight culture were transferred to fresh LB broth and grown until reaching an absorbance of 0.8 at 600 nm. Protein expression was induced with 0.2 mM IPTG, followed by overnight incubation at 20°C. The cells were collected and lysed using 50 ml of lysis buffer [30 mM Tris-HCl (pH 8.0), 20% sucrose, 1 mM EDTA, 0.3 mg/ml lysozyme, and 1 mM PMSF] and cell debris was removed by centrifugation. Precipitation with ammonium sulfate at 20% and 70% saturation was performed, followed by resuspension and desalting on a PD-10 column. The desalted proteins were passed through an ANTI-FLAG M2 affinity gel, washed [25 mM Tris-HCl buffer (pH 7.5)], and eluted [0.1 M glycine-HCl buffer (pH 3.5)]. The purified C-ter100 proteins were neutralized [1 M Tris-HCl buffer (pH 8.0)], concentrated, and stored at -20°C. To assess the endotoxin levels of the recombinant proteins used in this study, we utilized the ToxinSensor Chromogenic LAL Endotoxin Assay Kit from Genescript (New Jersey, USA). No endotoxins were detected in the purified recombinant proteins in this study.

### Purification of N-ter139 and preparation of anti-vEP antibody

The coding region for the N-terminal 139 amino acid stretch (named N-ter139) of vEP-45 was PCR-amplified from the genomic DNA of V. *vulnificus* ATCC29307 using the forward primer 5′-TCAGCCTTCGGAGCGCAAGCAGACGGCACTGGA-3′ and the reverse primer 5′-GACTCTAGAGGACAACGGATAGAAGGTAGACGC-3′. The underlined bases in the primers indicate the introduced restriction sites, EcoRI and XbaI, respectively. The PCR conditions involved 35 cycles of denaturation at 94°C for 30 s, annealing at 60°C for 30 s, and extension at 72°C for 40 s. The resulting PCR product was digested with EcoRI and XbaI and ligated with EcoRI/XbaI-digested pMAL-c2X vector (Addgene, Teddington, UK) to generate the recombinant plasmid, pN-ter139.

For N-ter139 protein expression, *E*. *coli* DH5α cells with pN-ter139 were cultured in LB medium with ampicillin until reaching an OD_600_ of 0.5. IPTG (0.3 mM) was added for induction, followed by incubation for 4 hours at 37°C. The cells were resuspended and sonicated several times for 30 seconds each in a lysis buffer [50 mM Tris-HCl (pH 7.5), 1 mM PMSF, 1 mM EDTA, 0.5 mM DTT, and 10% glycerol]. The protein suspension was applied to an amylose column, washed [25 mM Tris-HCl buffer (pH 7.5)], and eluted with maltose buffer [50 mM Tris-HCl (pH 7.5), 1 mM PMSF, 1 mM EDTA, 0.5 mM DTT, 10% glycerol and 10 mM maltose]. Eluted proteins were concentrated, aliquoted, and stored at -20°C. The purified N-ter139 protein was used as an antigen to produce polyclonal anti-N-ter139 antibody in rabbit by Peptron (Daejeon, Korea). Anti-vEP IgGs were purified from the rabbit sera using CNBr-activated Sepharose 4 column chromatography and used as anti-vEP antibody.

### ELISA and caspase-1 activity assay

RAW264.7 cells (1 × 10^5^ cells/well) were plated on 48-well plates and treated with 1 μg/ml of LPS, vEP-45, vEP-34, or C-ter100 for 3 h at 37°C. TNF-α concentration in the culture medium was measured using an ELISA kit (R&D System, Minneapolis, USA). BMDMs (1 × 10^5^ cells/well) on 48-well plates were treated similarly, followed by incubation with 2 mM ATP at 37°C for 2 h. IL-1β levels and caspase-1 activity in the culture media were measured using IL-1β ELISA kit (R&D System, Minneapolis, USA) and Caspase-Glo 1 inflammasome assay kit (Promega, Madison, WI, USA), respectively.

### RT-PCR

A total of 5 × 10^6^ cells were seeded onto 100-mm culture dishes and treated with 1 μg/ml of LPS, vEP-45, vEP-34, or C-ter100 for various durations (0 to 12 hours). Following treatment, total RNA was extracted using an easy spin column (iNtRON Biotechnology, Seongnam, Korea), and cDNA synthesis was performed with oligo-dT primer and M-MLV reverse transcriptase (Bioneer, Daejeon, Korea) according to the manufacturer’s instructions. The synthesized cDNAs served as templates for PCR amplification of the target genes. Specific primers and cycling conditions are detailed in S2 Table. Typically, PCR amplification was carried out with 0.5 μg of cDNA, 2.5 units of Taq DNA polymerase, 2.5 mM of each dNTP, and 10 pmol of specific forward and reverse primers in a PCR buffer [1.5 mM MgCl2, 50 mM KCl, 10 mM Tris-HCl (pH 8.3)]. The PCR conditions consisted of 30 cycles of denaturation at 94°C for 30 s, annealing at various temperatures depending on the primers for 30 s, and extension at 72°C for 40 s (S2 Table). The resulting PCR products were separated by electrophoresis on a 2% agarose gel, stained with EtBr, and visualized using a UV transilluminator. Band densities were quantified using the Scion Image program (Scion Corp., Frederick, MD, USA).

### Western blot analysis

Cells were lysed with a buffer (20 mM HEPES, pH 7.4, 1 mM EGTA, 1 mM EDTA, 10 mM NaCl, 1.5 mM MgCl_2_, 0.25% Triton X-100, 1 mM PMSF, 10 μg/ml leupeptin, 2 mg/ml pepstatin A, 2 mg/ml aprotinin, and 1 mM DTT) for 20 min and centrifuged at 15,000 ×g for 10 min at 4°C. Equal amounts of proteins (50 μg) were loaded onto a 12% or 15% SDS-polyacrylamide gel and transferred to PVDF membranes (BioRad, Hercules, California, USA). The membranes were washed with TBS-T buffer (20 mM Tris-HCl, pH 7.5, and 0.1% Tween 20) and blocked with a blocking buffer (5% non-fat milk in TBS-T) for 3 h at 20°C on a shaker. The membrane was incubated overnight at 4°C with specific antibodies in the blocking buffer, washed three times with TBS-T buffer, and reacted with secondary antibodies coupled to for 1 h at 20°C. After washing several times with the blocking buffer, the blot signals were detected with West-Zol plus detection kit (iNtRON Biotechnology, Sungnam, Korea) on X-ray film.

### Measurement of NO synthesis

Normally a total of 5 × 10^5^ cells was plated on 48-well plates and treated with 1 μg/ml of LPS, vEP-45, vEP-34, or C-ter100 for 18 h at 37°C. The amount of produced NO was estimated by measuring the nitrite concentration in the medium using a Griess assay [49].

### EMSA and super shift assay

RAW264.7 cells were exposed to 1 μg/ml of LPS, vEP-45, vEP-34, and C-ter100 for varying durations, then rinsed with PBS and suspended in lysis buffer (10 mM HEPES, pH 7.9, 0.5 mM KCl, 1.5 mM MgCl2, 0.5 mM DTT, and 0.2 mM PMSF). After incubation on ice for 5 minutes, the suspension was centrifuged at 15,000 xg for 5 minutes. The resulting pellet was reconstituted in a high salt buffer (20 mM HEPES, pH 7.9, 25% glycerol, 1.5 mM MgCl2, 0.8 M KCl, 0.2 mM EDTA, 0.5 mM DTT, 0.2 mM PMSF) and centrifuged at 15,000 xg for 20 minutes. Supernatants containing nuclear extracts were collected for EMSA and super shift assays. For EMSA, double-stranded oligonucleotides containing a consensus NF-κB binding site (5′- AGCTTGGGGACTTTCC-3′) were dephosphorylated with alkaline phosphatase and end-labeled for 10 min at 37°C with [γ-^32^P]-ATP (50 μCi at 3,000 Ci/mmol; Amersham Pharmacia Biotech Co. Uppsala, Sweden) and 5 U of T_4_ polynucleotide kinase. A total of 20 μl of reaction mixture composed of 10 μg of the nuclear extract, 0.5 pmol of the radiolabeled oligonucleotides, and 250 ng of poly (dI·dC) in a buffer (5% glycerol, 1 mM EDTA, and 1 mM DTT) was then prepared and incubated for 20 min at 20°C. The DNA-protein complexes formed were then separated on a native 6% polyacrylamide gel in 0.5% Tris-borate-EDTA buffer and autoradiographed on X-ray film to visualize. For super shift assay, 2 μg of anti-p65 antibody was further added to the reaction mixture, incubated at 4°C for 1 h, electrophoresed, and autoradiographed as in EMSA.

### Co-immunoprecipitation

RAW264.7 cells (1 x 10^6^ cells/dish) were seeded on 100 mm dish, cultured for 2 days, and harvested by centrifuging 1,000 x*g* at 4°C for 10 min. The cell pellet was resuspended in a lysis buffer [20 mM HEPES, pH 8.0, 100 mM NaCl, 1 mM EDTA, 1 mM PMSF, and 1% (v/v) Nonidet P-40] and incubated at 4°C for 1 h. The sample underwent centrifugation at 15,000 ×g for 20 minutes to eliminate insoluble proteins, and the resultant supernatant containing membrane proteins was gathered. For immunoprecipitation, 10 mg of membrane proteins prepared were incubated with 1 μg of vEP-45, vEP-34, or C-ter100 and precipitated with 1 μg of anti-TLR4 antibody coupled to protein A agarose (Merck, Darmstadt, Germany) at 4°C for 1 h. The immunoprecipitates were washed twice in the same lysis buffer and solved with non-reducing SDS sample buffer [0.5 M Tris-HCl (pH 6.8), glycerol, 10% SDS, β-mercaptoethanol, 0.5% bromophenol blue] by boiling for 5 min. The samples were then separated by 12% SDS-PAGE and the proteins were transferred to a PVDF membrane. Western blottings were performed with anti-vEP, anti-FLAG (Merck, Darmstadt, Germany) or anti-TLR4 antibodies (diluted to 1:1000; Santa Crize, Texas, USA) as described elsewhere.

### Immunofluorescence assays using confocal microscopy

RAW264.7 cells (1 x 10^6^ cells/well) cultured for 24 h on coverslips in 12 well plate were treated with 1 μg of LPS, vEP-45, vEP-34, or C-ter100 for 30 min at 37°C, washed with ice-cold PBS, and then fixed with 4% paraformaldehyde for 10 min. After blocking with 3% BSA in PBS for 1 hour, cells were immunostained overnight with preabsorbed anti-FLAG antibodies, followed by overnight staining with anti-TLR4 antibodies (diluted 1:50). Subsequently, the cells were stained for 1 hour with IgG antibodies conjugated to fluorescein or rhodamine. BMDMs (1 × 10^6^ cells/well) on coverslips in 12-well plates were treated with 0.1 μg of LPS, vEP-45, vEP-34, or C-ter100 for 3 h, then stimulated with 2 mM ATP for 2 h at 37°C. After fixing and blocking, cells were incubated overnight with a 1:100 dilution of anti-NLRP3 or -ASC antibody, followed by staining with fluorescein- or rhodamine-conjugated IgG antibody (1:200 dilution) in PBS for 1 h. Nuclei were stained with 4’, 6-diamidino-2-phenylindole (DAPI). Zeiss LSM 510 confocal microscope (Le Pecq, France) was used for observation.

### Design of RNAi molecules and plasmid construction

Based on the complete genome of TLR4 (GenBank Accession No. NM_021297), RNAi duplexes were designed and synthesized by Bioneer (Daejeon, Korea). This RNAi expression vector, pSilencer 4.1-CMVneo plasmid (Ambion, Austin, USA), was linearized with digestion with both BamH I and Hind III enzymes to facilitate directional cloning of the RNAi duplexes. The sense and the anti-sense strands of TLR4 RNAi were as follows: 5’-GATCCCGACTTACAGTTTCTACGTTTCAAGAGAA CGTAGAAACTGTAAGTCGTTA-3’ and 5’-AGCTTAACGACTTACAGTTTCTACGTTCTCTTGA AACGTAGAAACTGTAAGTCGG-3’, respectively [50]. The synthesized TLR-4 RNAi constructs were cloned into the BamH I/ Hind III-cut pSilencer 4.1-CMV neo plasmid, according to the manufacturer’s procedure.

### Transfection of RNAi constructs into RAW264.7 cells

A total of 1 × 10^6^ cells were plated on 24-well plates the day before RNAi transfection. RAW264.7 cells were transfected with 500 ng of TLR-4 RNAi plasmid by 1.25 μl of Lipofectamine 2000 (Invitrogen, Carlsbad, USA), according to the manufacturers protocol. The transfected cells were incubated at 37°C in a CO_2_ incubator for 24 h, and 600 μg/ml of G418 was added to obtain stable cell populations.

### Sample preparation of NMR titration

C-ter100 was cloned into the pET32a vector containing a Trx His-tag and TEV protease cleavage site in the N-terminus. For ^15^N isotope labeling, the protein was cultured in M9 media containing ^15^NH_4_Cl and ^12^C-glucose. Later, 1 mM IPTG was added when the cells reached an OD_600_ of 0.6, and the mix was incubated overnight at 25°C. After cell harvest, the cell pellet was lysed in 20 mM sodium phosphate pH 7.0, 300 mM NaCl, 5 mM DTT, and 1 mM protease inhibitor cocktail. After sonication and centrifugation, the supernatant was collected and loaded onto a Ni-NTA column. The column was washed twice using a washing buffer (20 mM sodium phosphate pH 7.0, 300 mM NaCl, 5 mM DTT, and 20 mM imidazole). Proteins were eluted with elution buffer (20 mM sodium phosphate pH 7.0, 300 mM NaCl, 5 mM DTT, and 300 mM imidazole). After desalting, TEV protease was added to the purified protein and incubated for 11 h at 25°C. After reverse column work, C-ter100 was loaded into the size-exclusion gel chromatograph in 20 mM HEPES, pH 8.0, 200 mM NaCl, and 0.01% NaN_3_. Pure isolated protein was concentrated to 0.2 mM and transferred into a 5-mm Shigemi tube.

A toll-like receptor 4 construct, named TLR4^TV3^, was obtained as a gift from Lee’s group, KAIST, Republic of Korea [51]. The TLR4^TV3^ gene sequence was cloned into the pAcGP67 vector containing an Fc-tag and a thrombin cleavage site, and the protein was overexpressed in Sf9 and secreted into CCM media. The protein was purified with a protein A Sepharose affinity column. The Fc-tag was removed after the thrombin cleavage reaction. NEXT, the isolated protein was further purified by ion-exchange column and size-exclusion gel chromatography. The final buffer conditions were 20 mM HEPES, pH 8.0, 200 mM NaCl. The TLR4^TV3^ molecular weight of 31 kDa was confirmed by SDS-PAGE. The purified protein was concentrated up to 3 mg/ml (0.1 mM).

### NMR titration

NMR titration was performed with a Bruker DRX900 spectrometer equipped with a cryogenic probe head. ^1^H, ^15^N-Heteronuclear single quantum coherence (HSQC) was collected at 298 K and all data were processed using NMRPipe and NMRDraw. The NMR spectra were analyzed with the SPARKY program (version 2.3.0).

### Molecular modeling

A refined docking model was built up using the AutoDock program based on the NMR titration results. The crystal structure of TLR^TV3^ (PDB ID: 4G8A) was used as a receptor template and the active and passive residues were not assigned during molecular docking. Seven residues (i.e., Asp553, Tyr574, Asn577, Ile594, Lys595, Tyr597, and Asn599) were classified as active residues of the ligand molecule, C-ter100, and the passive residues near the active residues were assigned automatically. The overall lowest energy structure was used for further molecular analysis.

### Neutralizing antibody assay

To make C-ter100Mab, 1 mg of C-ter100 was purified by previously mentioned method and a mouse monoclonal antibody against C-ter100 was requested by Zenbody Inc. (Cheonan, Korea). Serial dilutions of C-ter100Mab (0.1, 0.05, 0.025, and 0.01 mg/ml) and C-ter100 (1, 0.5, 0.01, and 0.005 μg/ml) were obtained and mixed in various combinations for 1 h at 20°C, to determine the appropriate titrating neutralizing efficiency. The rest of the experiments were performed with C-ter100Mab (0.25 mg/ml) and C-ter100 (0.1 μg/ml) pre-incubated at 20°C for 1 h.

### Flow cytometry and histamine release

At 8 weeks of age, BALB/c mice received injections of 2.5 mg/kg each of LPS and vEPs (vEP-45, vEP-34, and C-ter100) via the tail vein. Three hours post-injection, whole blood was obtained via retro-orbital bleeding using K2-EDTA-coated tubes (BD Vacutainer, Becton Dickinson, NJ, USA) with reactions halted by 1 mM of 1,10-PT addition. One hundred microliters of whole blood was dispensed into 5 ml polystyrene round-bottom test tubes (Falcon, BD Labware, NJ, USA). Subsequently, 20 μl of human Fc receptor binding inhibitor (eBioscience, San Diego, USA) was added, and the mixture was incubated on ice for 15 minutes to block Fc receptors. Antibodies targeting Ly-6G-PE, Ly-6C-PerCP, and CD11b-APC (eBioscience, San Diego, USA) were added at recommended concentrations by the manufacturer in a total volume of 50 μl for 25 min at 20°C in the dark. Subsequently, 2 ml of 1× RBC lysing solution (eBioscience, San Diego, USA) were added to lyse red blood cells, followed by a 20-min dark incubation at 20°C. Following centrifugation for 5 minutes at 500 × g at 20°C, the supernatant was removed, and the cells were rinsed with 2 ml of flow cytometry staining buffer (eBioscience, San Diego, USA) before being suspended in 200 μl of the same buffer. FACS analysis was carefully optimized and conducted by facility managers with appropriate controls using a FACS-Calibur instrument (Becton Dickinson, USA), with data analysis conducted using WinMDI 2.8 software. Each experimental group comprised six animals.

To measure the concentration of released histamine, 2 ml of whole blood were aliquoted in 5 ml round polystyrene bottom test tubes and centrifuged for 10 min at 1,500 ×*g*. The concentration of histamine in plasma was measured using an ELISA kit (Enzolifesciences, NY, USA), according to the protocols provided by the manufacturer. Each group included six animals.

## Supporting information

S1 Table Purification summary for C-ter100 and N-ter139. (DOCX)

S2 Table RT-PCR primer sequences and cycling conditions. (DOCX)

S1 Fig Transcription levels of pro-inflammatory cytokines and inflammatory regulators by vEP-45 and C-ter100. (A-D) Effects of transcription levels of pro-inflammatory cytokines. RAW264.7 cells were treated with LPS, vEP-45, vEP-34, and C-ter100 (1 μg/ml) for the indicate time periods and RT-PCR was performed using specific primers for *Tnf-α*, *Il-1β*, *Il-6*, and *Gapdh* as an internal control. The resulting PCR products were visualized on 1.2% agarose gel and stained with EtBr (A). Quantification of band intensities at 3 h of *Tnf-α* (B), *Il-1β* (C), and *Il-6* (D) was conducted from two independent experiments, normalized with those of *Gapdh*, and expressed as the mean (±SD) values in fold, compared those of corresponding none-treated groups experiments. (E-I) Effects of transcription levels of inflammatory regulators. RAW264.7 cells were treated with 1 μg/ml each of LPS, vEP-45, vEP-34, and C-ter100 for the time periods indicated and RT-PCRs were performed with primers specific for *Cox-2*, *Pges*, *iNos*, *Mip-2*, and *Gapdh* as an internal control. The resulting PCR products were subjected on 1.2% agarose gel and stained with EtBr to visualize (E). The band intensities at 3 h of *Cox-2* (F), *Pges* (G), *iNos* (H), and *Mip-2* (I) were measured from two-independent experiments, normalized with those of *Gapdh*, and expressed as the mean (±SD) values in fold, compared those of corresponding none-treated groups experiments (*, *p* < 0.005; **, *p* < 0.01; significant differences, compared to that of non-treated sample using one-way ANOVA, followed by Dunnett’s multiple comparison test).
(TIF)

S2 Fig Effect of anti-C-ter100 monoclonal antibody on the production of TNF-α. Raw264.7 cells were treated with 1 μg/ml each of LPS, vEP-45, vEP-34, and C-ter100 for 3 h in the absence or presence of anti-C-ter100 monoclonal antibody (C-ter100Mab) and mouse IgG, from which TNF-α concentrations in the culture supernatant were measured using ELISA and expressed as the relative production, compared to the amount of TNF-α produced by C-ter100 as 100%. Data represent the mean value ±SD of duplicate determinations from three different experiments (*, *p* < 0.001; significant differences compared to non-treated sample using one-way ANOVA, followed by Dunnett’s multiple comparison test. ^ǂ^, *p* < 0.001; *p****-***value was obtained using a paired *t*-test, compared with each indicated group).
(TIF)

S1 Source Data Source data for Figs 1–3 and S1 and S2 Figs. (XLSX)
