# Supplementary material for: C-ter100 peptide derived from Vibrio vEP-45 protease acts as a pathogen-associated molecular pattern to induce inflammation and innate immunity
Source: PLoS Pathog. 2024 Aug 26;20(8):e1012474. doi: 10.1371/journal.ppat.1012474 (PMC11379387; doi:10.1371/journal.ppat.1012474)
Supplement: S1 Table — (DOCX) [file ppat.1012474.s001.docx]

**S1 Table. Purification summary for C-ter100 and N-ter139**

| **Protein** | **Purification step** | **Total protein (mg)** | **Yield (%)^a^** |
| --- | --- | --- | --- |
| C-ter100 | Periplasmic protein | 130 | 100 |
|  | FLAG agarose | 2.32 | 1.8 |
| N-ter139 | Cell extract | 131.5 | 100 |
|  | Amylose column | 25.5 | 19 |

^a^ The initial purification step was designated as having 100% total activity
