# Supplementary material for: C-ter100 peptide derived from Vibrio vEP-45 protease acts as a pathogen-associated molecular pattern to induce inflammation and innate immunity
Source: PLoS Pathog. 2024 Aug 26;20(8):e1012474. doi: 10.1371/journal.ppat.1012474 (PMC11379387; doi:10.1371/journal.ppat.1012474)
Supplement: S2 Table — (DOCX) [file ppat.1012474.s002.docx]

S2 Table. RT-PCR primer sequences and cycling conditions

| **Gene** | | **Primer sequence**  **(5’ to 3’)** | **Expected size**  **(bp)** | **Annealing temperature**  **(℃)** | **GeneBank**  **Accession No.** |
| --- | --- | --- | --- | --- | --- |
| *Tnf-α* | Forward | TTTCCTCCCAATACCCCTTC | 197 | 48 | Y00467 |
|  | Reverse | AGTGCAAAGGCTCCAAAGAA |  |  |  |
| *Il-1β* | Forward | TGTGAAATGCCACCTTTTGA | 205 | 50 | AK168047 |
|  | Reverse | GTAGCTGCCACAGCTTCTCC |  |  |  |
| *Il-6* | Forward | AATTTCCTCTGGTCTTCTGG | 220 | 50 | M24221 |
|  | Reverse | TAGCCACTCCTTCTGTGACTC |  |  |  |
| *Cox-2* | Forward | CAGCAAATCCTTGCTGTTCC | 658 | 50 | BC052900 |
|  | Reverse | CCATCCTTGAAAAGGCGCAG |  |  |  |
| *iNos* | Forward | GTGTTCCACCAGGAGATGTTG | 576 | 53 | BC062387 |
|  | Reverse | CTCCTGCCCACTGAGTTCGTC |  |  |  |
| *Pges* | Forward | ATGCCTTCCCCGGGCCTG | 462 | 48 | AK150013 |
|  | Reverse | TCACAGATGGTGGGCCAC |  |  |  |
| *Mip-2* | Forward | AACAAAGGCAAGGCTAACTGA | 203 | 53 | X53798 |
|  | Reverse | AACATAACAACATCTGGGCAAT |  |  |  |
| *Tlr4* | Forward | ATACATTCCTGTAAGTTACCTG | 546 | 48 | NM_021297 |
|  | Reverse | CTGCTTAAGTTGACATCTAATGAT |  |  |  |
| *Gapdh* | Forward | TCAGCAATGCATCCTGCACCAC | 252 | 55 | BC082592 |
|  | Reverse | TGCCAGTGAGCTTCCCGTTCAG |  |  |  |
