# Supplementary figures and images for: C-ter100 peptide derived from Vibrio vEP-45 protease acts as a pathogen-associated molecular pattern to induce inflammation and innate immunity
Source: PLoS Pathog. 2024 Aug 26;20(8):e1012474. doi: 10.1371/journal.ppat.1012474 (PMC11379387; doi:10.1371/journal.ppat.1012474)

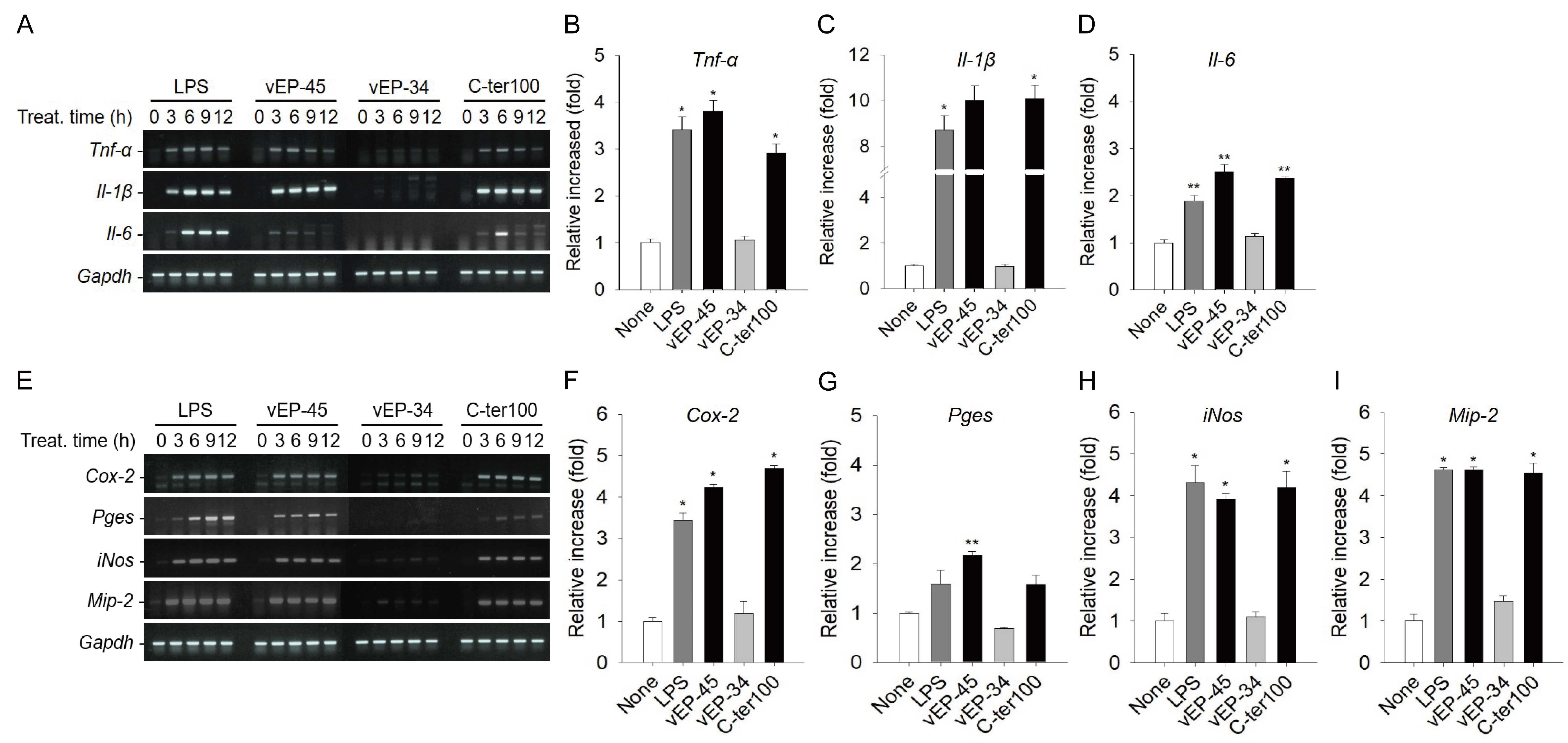

Supplement: S1 Fig — (A-D) Effects of transcription levels of pro-inflammatory cytokines. RAW264.7 cells were treated with LPS, vEP-45, vEP-34, and C-ter100 (1 μg/ml) for the indicate time periods and RT-PCR was performed using specific primers for Tnf-α, Il-1β, Il-6, and Gapdh as an internal control. The resulting PCR products were visualized on 1.2% agarose gel and stained with EtBr (A). Quantification of band intensities at 3 h of Tnf-α (B), Il-1β (C), and Il-6 (D) was conducted from two independent experiments, normalized with those of Gapdh, and expressed as the mean (±SD) values in fold, compared those of corresponding none-treated groups experiments. (E-I) Effects of transcription levels of inflammatory regulators. RAW264.7 cells were treated with 1 μg/ml each of LPS, vEP-45, vEP-34, and C-ter100 for the time periods indicated and RT-PCRs were performed with primers specific for Cox-2, Pges, iNos, Mip-2, and Gapdh as an internal control. The resulting PCR products were subjected on 1.2% agarose gel and stained with EtBr to visualize (E). The band intensities at 3 h of Cox-2 (F), Pges (G), iNos (H), and Mip-2 (I) were measured from two-independent experiments, normalized with those of Gapdh, and expressed as the mean (±SD) values in fold, compared those of corresponding none-treated groups experiments (*, p < 0.005; **, p < 0.01; significant differences, compared to that of non-treated sample using one-way ANOVA, followed by Dunnett’s multiple comparison test). (TIF) [file ppat.1012474.s003.tif]

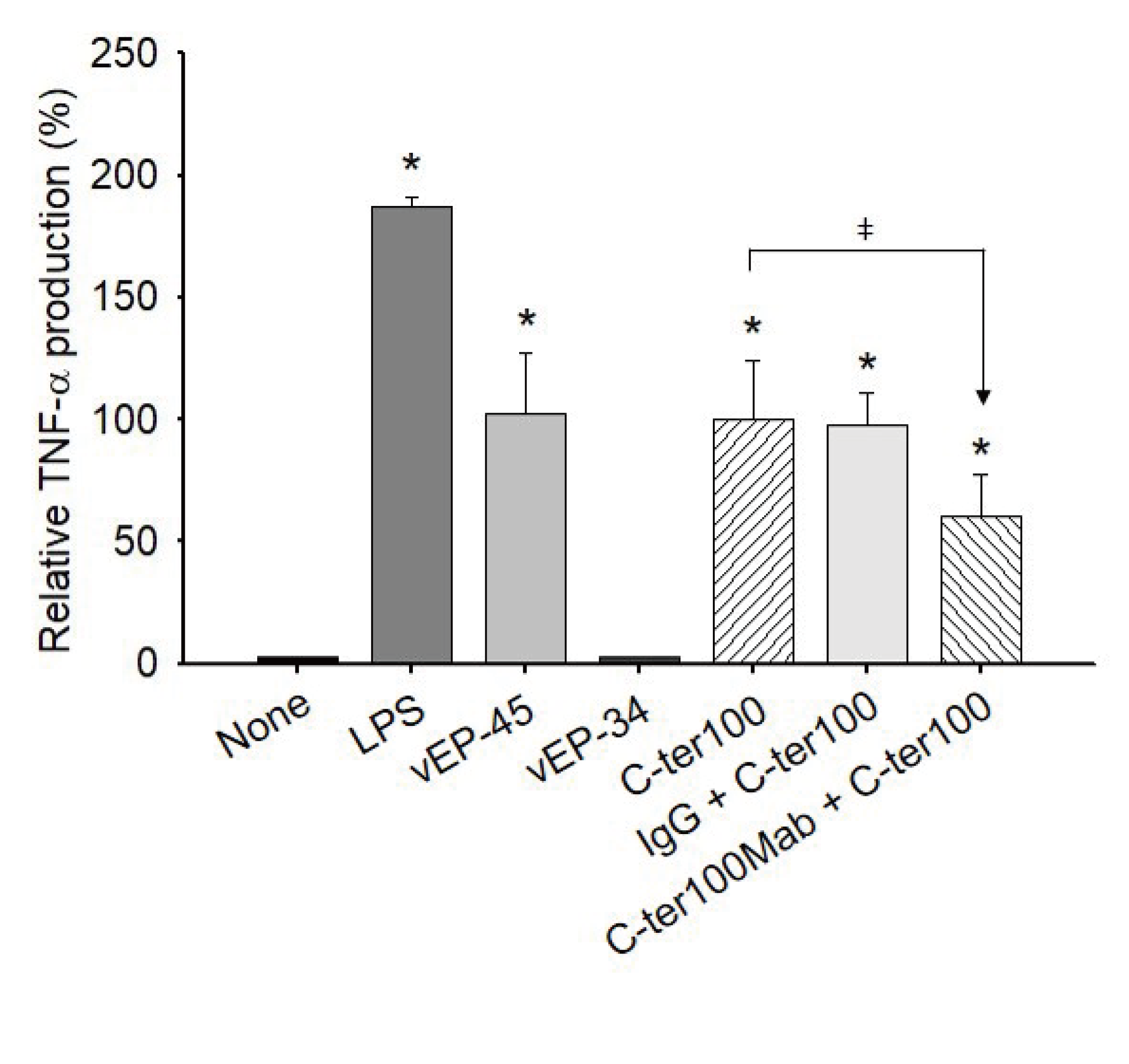

Supplement: S2 Fig — Raw264.7 cells were treated with 1 μg/ml each of LPS, vEP-45, vEP-34, and C-ter100 for 3 h in the absence or presence of anti-C-ter100 monoclonal antibody (C-ter100Mab) and mouse IgG, from which TNF-α concentrations in the culture supernatant were measured using ELISA and expressed as the relative production, compared to the amount of TNF-α produced by C-ter100 as 100%. Data represent the mean value ±SD of duplicate determinations from three different experiments (*, p < 0.001; significant differences compared to non-treated sample using one-way ANOVA, followed by Dunnett’s multiple comparison test. ǂ, p < 0.001; p-value was obtained using a paired t-test, compared with each indicated group). (TIF) [file ppat.1012474.s004.tif]
